# Elbow fractures: from fixation to replacement

**DOI:** 10.1186/1753-6561-9-S3-A85

**Published:** 2015-05-19

**Authors:** Amit Gupta

**Affiliations:** 1 Department of Orthopedic Surgery, University of Louisville, Kentucky, 40292, USA

## 

Distal humerus fractures may range from a simple extra-articular fracture pattern to a complex pattern, involving extensive destruction of the articular surface with comminution and bone loss in the metaphyseal-diaphyseal junction of the bone.

The management decision in the younger patient requires complete anatomical reconstruction and early active range of motion. Different approaches to the distal humerus will be discussed, as will be different fixation methods. I will also present relevant clinical cases and outline the known outcomes of these fractures.

In the elderly with osteoporotic fractures especially those with preexisting arthritis, there is an emerging trend for primary total elbow replacement. The outcomes of such replacement have been shown to be comparable to those of total elbow replacement in arthritis. However, there have been a few complications reported in the literature and this method is best reserved for complex comminuted distal humerus fractures in patients with significant osteoporosis and those above the age of 70.

I will also discuss complex fracture dislocations of the elbow joint in terms of the management decisions and outcomes of these difficult problems.

